# Typologies of dependency, household characteristics, and disparity in formal and informal care use: analysis of community-dwelling long-term care insurance claimants in an urban municipality of China

**DOI:** 10.1186/s12939-023-02048-5

**Published:** 2023-11-10

**Authors:** Shuai Fang, Hong Liang, Yan Liang

**Affiliations:** 1 Institute of Sociology, Shanghai Academy of Social Sciences, 622 Huaihai Middle Rd., Huangpu District, Shanghai, 200020 China; 2 School of Social Development and Public Policy, Fudan University, 220 Handan Rd., Yangpu District, Shanghai, 200433 China; 3 School of Nursing, Fudan University, 305 Fenglin Rd., Xuhui District, Shanghai, 200032 China

**Keywords:** Cluster analysis, Dependency, Household characteristics, Long-term care use, Socioeconomic inequality

## Abstract

**Background:**

A comprehensive understanding of subgroups of community-dwelling older adults and their long-term care (LTC) utilization can help to promote equality in the long-term services and support system. Dependency and household characteristics were found to affect the LTC utilization of homebound older adults. However, few studies considered the typologies of dependency of older populations according to co-occurring limitations, and little is known about differences in LTC use among elderly of typologies of dependency under distinct household conditions.

**Methods:**

We aimed to identify typologies of dependency of older adults living at home and explore the disparities in formal care and informal care use among typologies of dependency by income and living situation. In this cross-sectional study, we used the public long-term care insurance (LTCI) database of Yiwu, Zhejiang Province, China, and included 1675 individuals aged ≥ 60 years living at home. Cluster analysis was conducted to determine typologies of dependency among older adults. A two-step multilevel analysis was used to examine disparities in formal and informal care use related to household income and living status among typologies of dependency.

**Results:**

Seven dependency clusters were identified. Pro-wealthy inequalities in both formal and informal care use were found in the least dependent cluster and the limited-locomotion cluster. Pro-poor inequalities in formal care use were found in the fully dependent cluster without impaired vision and the cluster with intact continence and vision. Living with family members was positively associated with receiving formal care for the fully dependent cluster. Older adults in most clusters were more likely to use informal care when living with family members, except for the least dependent cluster and the limited-locomotion cluster.

**Conclusions:**

Our findings suggest that household inequalities in LTC use varied among typologies of dependency of older adults, which may provide insights for researchers and policymakers to develop tailored LTC and targeted LTCI programs for older adults living at home and their family caregivers, considering both typologies of dependency and household characteristics.

## Introduction

Population aging is one of the current challenges in most countries, putting enormous pressure on health and social security systems [1]. Such demographic changes might also imply a growing population with multimorbidity and decreasing functional capacity, requiring assistance for daily living activities in formal (paid) and informal (unpaid) care [2]. It is predicted that by 2030, the number of people over the age of 60 in China who will require care due to disability will reach 138 million, 14.02 million more than in 2020 [3]. The Chinese government is committed to establishing a national public long-term care insurance (LTCI) program. Fifteen cities in China implemented LTCI policies as the first pilot cities in 2016, with 34 more pilot cities to be included in 2020. At this stage, the target population is mainly elderly people with physical disabilities, but in the future it will be expanded to include people with intellectual disabilities and cover all age groups [4].

Dependency is a key individual determinant for long-term care (LTC) use [5]. Dependency, also often referred to as care dependency, is “a state of” and the core of this state is a “need,” which makes the person dependent on another person [6]. Dependency means that people require social, family or institutional support, due to temporary or definitive loss of their abilities [7]. Previous studies have typically measured dependency in terms of disability severity, such as the number of limitations in activities of daily living [7] or the score on composite scales [8]. These approaches might insufficiently capture larger clusters and qualitative traits related to an individual’s complex functional status. Because disability develops over time based on the accumulation and co-evolution of a range of typical impairments [9, 10], older adults tend to have multiple limitations at the same time [11]. Earlier studies have used functional limitation classes to describe patterns of functional decline, categorizing older adults into subgroups consisting of combinations of limitations [12]. However, little is known about typologies of dependency among older adults with physical disabilities who require LTC.

Person-oriented analyses, such as cluster analysis or latent class analysis (LCA), which use patterns of scores across cases to identify individuals who can be grouped together [13], provide opportunities to explore the complexity of functional limitations and to identify typologies of dependency among older adults. Cluster analysis and LCA make different assumptions about the data; cluster analysis assumes that the cases with the most similar scores across the analysis variables belong in the same cluster, while LCA assumes that latent classes exist and explain patterns of observed scores across cases [13]. In addition, analysis variables in cluster analysis should be continuous, while the analysis variables in LCA are categorical [13]. In this study, because we used LTC claimants data, in which the dependency parameters were continuous and contributed jointly to the results of the LTC eligibility assessment (which means that the dependency parameter scores were similar and comparable), cluster analysis was more appropriate in this study for identifing typologies of dependency among older adults with physical disabilities who require LTC.

Notably, understanding the characteristics of long-term care (LTC) use is critical to estimating society’s demand for and costs of services [14]. The variation in LTC use among disabled older adults has been widely studied [15, 16]. Moderate and severe dependence has been found to be significantly associated with older people’s greater use of formal and informal care services, regardless of their individual and household characteristics; this has been suggested to be related to inequalities in LTC access [17, 18]. Much attention has also been given to household characteristics, which affect the LTC resources available to dependent elderly people to a large degree [17, 19]. Socioeconomic gradients of household income are generally described in terms of diverse LTC service utilization. For example, intensive informal care is concentrated among individuals with lower socioeconomic levels, and formal services are concentrated among those with higher socioeconomic levels [20]. More precisely, the higher the household income, the lower the use of only formal and informal care, and the higher the receipt of mixed care [21, 22]. Living status affects inequalities in the use of LTC services as it can determine access to informal care or the ability to count on potential “advocates” in receiving public social services [23–26]. However, studies on LTC use rarely take into account subpopulation-level differences resulting from different typologies of dependency, especially in view of different household characteristics (such as household income and living status). This lack of understanding will limit targeted subpopulation interventions and policies to reduce inequalities in LTC access.

From a policy perspective, care time is often used to estimate care costs and the burden on family caregivers, as well as to justify access to nursing homes, support staff, and financing by older people with disability, as it represents the service intensity related to the difficulty with providing assistance for elderly disabled adults and implies individual functional traits to a larger extent [27–29]. However, with respect to determinants of LTC use, the frequency of care reception [14] or whether the individual receives LTC [15] are often discussed.

Thus, in this study, we aimed: (1) to identify typologies of dependency among older adults with physical disabilities who require LTC, using public LTCI claimant data of 1675 older people in the city of Yiwu, Zhejiang Province, China; and (2) to examine disparities in formal and informal care use times on cluster level (subgroups of typologies of dependency), by income and living situation. The results of this study will offer a nuanced understanding of household socioeconomic inequality in LTC use among subgroups of typologies of dependency, and will be helpful for researchers and policymakers to improve care service provision and insurance payment policies for older adults through considering their functional limitation typologies as well as household conditions.

## Methods

### Data sources and participants

For this cross-sectional study, we obtained data from the public LTCI database of Yiwu in Zhejiang Province, China. As the key contact city in China’s National Long-term Care Insurance Pilot Project, Yiwu had a total of 1.07 million insured permanent residents as of 2018, and 10% of them were aged ≥ 60 years. Yiwu LTCI was initiated in September 2018, and the target population was adults aged 60 years. A set of standardized assessments was administered by trained professionals who visited claimants’ homes or facilities to determine the qualifications for being an LTCI beneficiary. Eligible older adults were ≥ 60 years and received the qualification assessment between 1 September and 31 December 2018 [4]. We included older adults living at home, and 1675 older people were finally included in the analysis. The mean age of the sample was 78.2 (standard deviation [SD] = 9.37) years, 49.3% (n = 826) of the sample was female, and 82% (n = 1373) were married.

### Variables

#### Dependent variables

In this study formal care use time and informal care use time were two dependent variables, and were assessed using two self-reported items, respectively: “In the past 3 months, how many hours per month, on average, did you use paid long-term care?” “In the past 3 months, how many hours per month, on average, did you use unpaid long-term care?” Hours of use of both formal and informal care were log-transformed.

#### Focal Independent variables

We focused on two aspects of individual determinants: typologies of dependency and household characteristics. (1) Typologies of dependency. We chose 19 items to identify typologies of dependency among participants, involving an assessment of mobility, self-care, urinary and fecal continence, locomotion, vision, and money management based on prior literature [30] and the dataset. The measurement level of each item was assessed using 1–5 levels of dependency, ranging from 1 (not needing assistance) to 5 (needing full assistance). (2) Household characteristics. In this study, we used household income and living status, which are widely used indicators to represent household characteristics. Household income was used as a binary variable and coded as low income = 1 and other = 0. Low income in the dataset represented those who were certified as a low-income population by the local government. Living status was created as a dichotomized variable based on its distribution (living with family members = 1, other = 0). This variable indicates the family structure which is considered to be the only informal support network characteristic consistently associated with the use of informal care services [31].

#### Confounders

Age, sex, marital status, and educational attainment were considered confounders and were self-reported, as follows: (1) age (years); (2) sex (male = 0, female = 1); (3) marital status (married = 0, and other = 1); and (4) educational attainment (illiterate or primary school = 0, middle school or higher = 1). In this study, self-rated health (SRH) was measured by the following single question: “How do you feel about your overall health, looking at the recent seven days?” This single-item questionnaire is considered a validated measure to reflect SRH and has been widely used in previous studies [32].Possible responses include very good, good, fair, poor, or very poor. This item was reverse-scored from 1 (very poor) to 5 (very good) so that higher scores reflected better SRH.

### Statistical analyses

Cluster analysis was used to identify typologies of dependency among elderly participants (for aim 1). K-means clustering was performed and the optimal number of clusters was determined based on the elbow method [33], as well as the percentage of between-group sum-of-squares (SS) in the total SS. An “elbow” or bend is the point where the total within the SS begins to level off. The higher the value of between_SS / total_SS, the better the clustering result, as this means a smaller within-group difference and a larger between-group difference.

For aim 2, first, one-way analysis of variance and cross tabulation were used to investigate between-cluster differences in sociodemographic variables, self-care health score, formal and informal use time, and additionally, by different income groups and living status groups. Then, we used two-step multilevel analysis to examine disparities in formal and informal care use times on cluster level (subgroups of typologies of dependency), by income and living situation, adjusting for confounders as mentioned above. *Twostep* is a bundle of programs to ease multilevel analyses with the “twostep approach”, and is easily applied in Stata by command *twostep* [34]. We used the two-step approach to multilevel analysis to estimate a parameter of interest in a unit-level dataset (individuals within subgroups of typologies of dependency) that is fed as a dependent variable into an analysis on the cluster level (subgroups of typologies of dependency). We performed log transformation of each individual’s age given its distribution and used the standardized variable in the regression models. All analyses were performed with R (The R Project for Statistical Computing, Vienna, Austria) and Stata SE 17.0 (StataCorp LLC, College Station, TX, USA).

## Results

### Participant characteristics

Table 1 presents the characteristics of the study participants. Among 1675 older adults, 29.4% (n = 493) were ≥ 85 years old; 32.3% (n = 540) were between 75 and 84 years old. Furthermore, nearly half (49.3%, n = 826) of participants were female, and 82% (n = 1373) were married. As for educational levels, 51.1% (n = 856) of participants were illiterate and 30.6% (n = 512) had graduated from primary school. Those who reported a low income level accounted for 13.3% (n = 223), and 76.6% (n = 1283) of participants lived with family members. The mean self-rated health score was 2.2 (SD = 0.90). The mean informal care time was 274.5 (SD = 238.5) hours per month, and the mean formal care time was 67.0 (SD = 183.9) hours per month.

**Table 1:** Participants’ characteristics and group differences (*N* = 1675)

Variable^†^		Cluster A	Cluster B	Cluster C	Cluster D	Cluster E	Cluster F	Cluster G	*P* value
	Whole sample (n = 1675)	Fully dependent without impaired vision (n = 299)	Fully dependent (n = 166)	Intact vision and transfer (n = 254)	Intact continence and vision (n = 305)	Able to groom (n = 132)	Limited locomotion (n = 298)	Least dependent (n = 221)	
	n (%) or M ± SD	n (%) or M ± SD	n (%) or M ± SD	n (%) or M ± SD	n (%) or M ± SD	n (%) or M ± SD	n (%) or M ± SD	n (%) or M ± SD	
**Age (years)**									< 0.001
60 − 74	642 (38.3)	106 (35.5)	43 (25.9)	69 (27.2)	117 (38.4)	57 (43.2)	142 (47.7)	108 (48.9)	
75 − 84	540 (32.3)	102 (34.1)	61 (36.8)	82 (32.3)	95 (31.1)	35 (26.5)	93 (31.2)	72 (32.6)	
≥85	493 (29.4)	91 (30.4)	62 (37.3)	103 (40.5)	93 (30.5)	40 (30.3)	63 (21.1)	41 (18.5)	
Female	826 (49.3)	152 (50.8)	82 (49.4)	124 (48.8)	151 (49.5)	61 (46.2)	151 (50.7)	105 (47.5)	0.970
Married	1373 (82.0)	251 (84.0)	125 (75.3)	207 (81.5)	263 (86.2)	114 (86.4)	243 (81.5)	170 (76.9)	0.019
**Education**									0.150
Illiterate	856 (51.1)	155 (51.8)	99 (59.6)	139 (54.7)	146 (47.9)	69 (52.3)	154 (51.7)	94 (42.5)	
Primary school	512 (30.6)	98 (32.8)	39 (23.5)	72 (28.4)	95 (31.1)	39 (29.5)	90 (30.2)	79 (35.8)	
Middle school or higher	307 (18.3)	46 (15.4)	28 (16.9)	43 (16.9)a	64 (21.0)	24 (18.2)	54 (18.1)	48 (21.7)	
Low income	223 (13.3)	36 (12.0)	17 (10.2)	33 (13.0)	37 (12.1)	15 (11.4)	42 (14.1)	43 (19.5)	0.125
Living with family members	1283 (76.6)	215 (71.9)	144 (86.7)	210 (82.7)	233 (76.4)	104 (78.8)	224 (75.2)	153 (69.2)	< 0.001
Self-rated health	2.2 ± 0.90	2.0 ± 0.88	1.8 ± 0.80	2.2 ± 0.82	2.2 ± 0.80	2.4 ± 0.98	2.3 ± 0.90	2.6 ± 0.93	< 0.001
Informal care time (hours per month)	274.5 ± 238.5	242.0 ± 235.2	392.8 ± 277.9	265.1 ± 217.8	299.8 ± 243.8	245.4 ± 224.0	265.3 ± 227.7	235.5 ± 219.3	< 0.001
Formal care time (hours per month)	67.0 ± 183.9	116.2 ± 222.8	111.4 ± 245.0	61.2 ± 169.8	85.8 ± 213.6	38.9 ± 152.8	22.1 ± 94.9	24.9 ± 111.4	< 0.001

*Note*: One-way ANOVA Bonferroni correction post-hoc tests were significant with *P* < 0.05 (correction already included). Chi-square post-hoc bivariate tests were significant with *P* < 0.007 (Bonferroni correction). ^†^One-way ANOVA was used for continuous variables, and the chi-square test was used for categorical variables to explore differences among groups

### Cluster analysis results

**Fig. 1:**
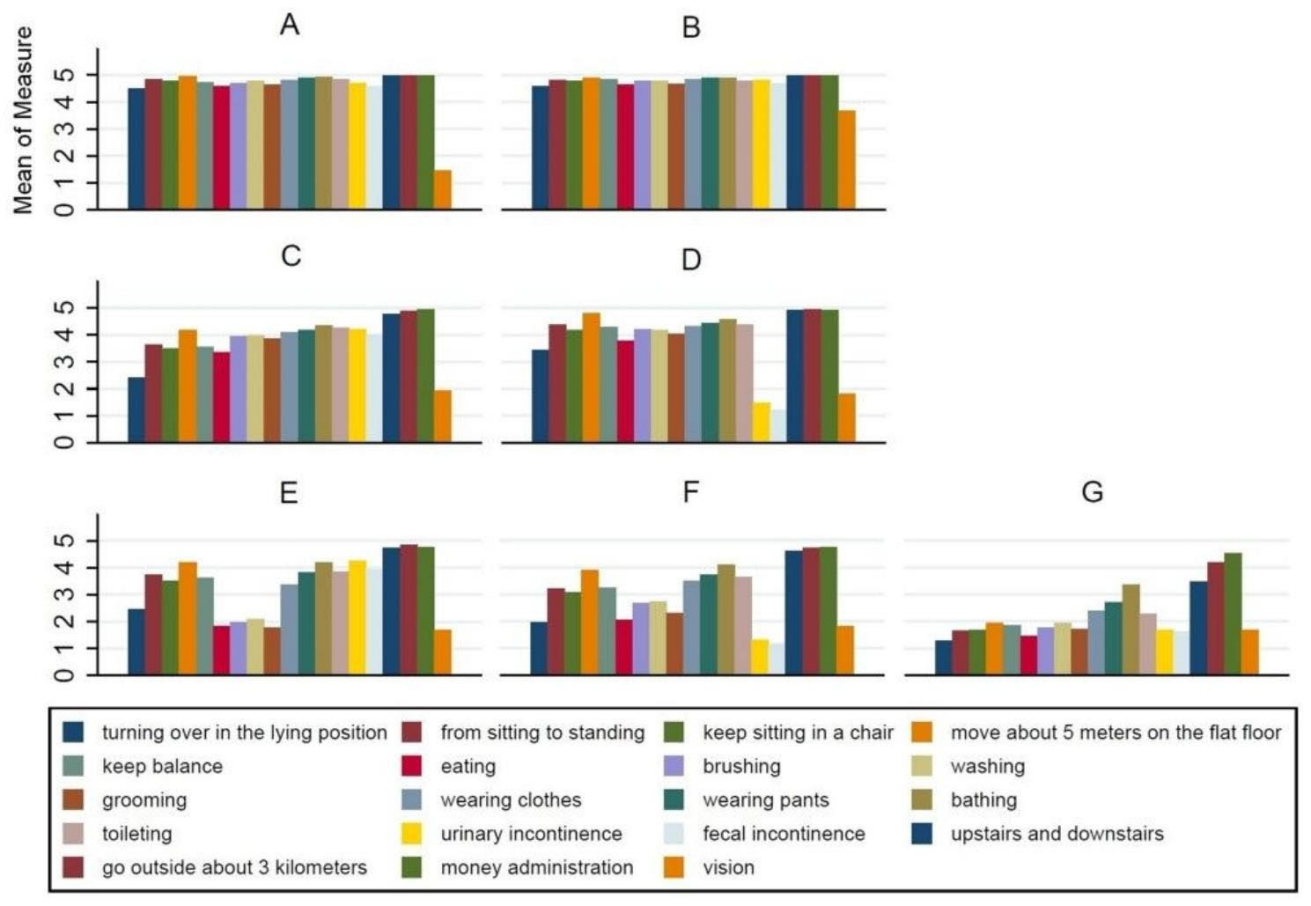
Seven-cluster grouping of LTCI claimants in Yiwu according to levels of 19 dependency parameters *Note*: **A**: fully dependent without impaired vision; **B**: fully dependent; **C**: intact vision and transfer; **D**: intact incontinence and vision; **E**: able to groom; **F**: limited locomotion; **G**: least dependent

Figure 1 shows the results of the cluster analysis. The optimal number of clusters was seven, as there was an “elbow” at the seven-cluster solution. The seven-cluster model yielded the larger percentage of between-group SS in the total SS (59.3%), compared with other models with several clusters from 4 to 6 (51.5%, 55.0%, 57.3%) and approaching the eight-cluster model (60.0%). Seven dependency clusters were identified (Fig. 1): fully dependent without impaired (17.9%), participants limited in all dependency parameters except in vision; fully dependent (9.9%), participants with dependency in all parameters; intact vision and transfer (15.2%), participants limited in nearly all dependency parameters except vision and transfer; intact continence and vision (18.2%), participants with dependency in nearly all parameters except incontinence and vision impairment; able to groom (7.9%), participants who performed well in eating, brushing, washing, and grooming; limited locomotion (17.8%), participants with dependency in locomotion and money management; and least dependent (13.2%), participants who performed well in self-care and mobility.

### Group comparisons among clusters

Table 1 shows the grouping comparisons among the seven clusters. Significant differences were observed in age, marital status, living status, self-rated health, and formal and informal care use. Participants in the least dependent cluster and limited -locomotion cluster were younger than those in other clusters. Participants in the fully dependent cluster and the cluster with intact vision and transfer were more likely to live with family members. Participants in the fully dependent cluster and the cluster with intact continence and vision were more likely to use informal care. The fully dependent without impaired vision cluster and the fully dependent cluster had the most formal care time. The least dependent cluster had the least time using either formal or informal care per month. In particular, the fully dependent without impaired vision cluster seemed to utilize less informal care time.

**Fig. 2:**
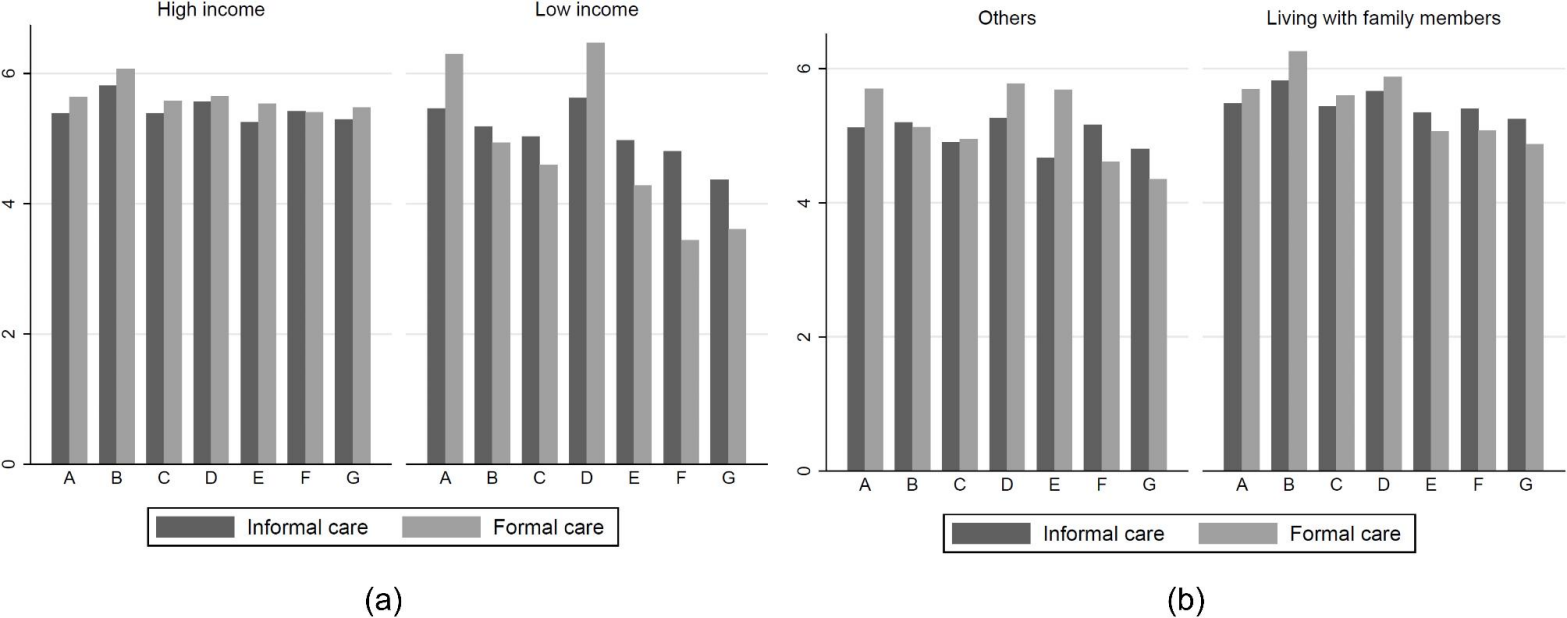
Informal and formal care use times among seven clusters by income and living situation

Figure 2 shows informal and formal care use in the seven clusters under distinct income levels and living statuses. Among older adults with high income levels, the cluster differences were small for both formal and informal care use time. For older adults in low income households, the cluster with intact continence and vision as well as the fully dependent without impaired vision cluster had the most time use both in formal and informal care. Comparatively, the fully dependent cluster and the cluster with intact vision and transfer had much less formal care time. Among older adults living with family members, those in the fully dependent cluster had the most formal care and informal care time. Among older adults in other living statuses, those in the cluster with intact continence and vision had the most informal and formal care; comparatively, the fully dependent cluster and intact vision and transfer cluster had much less formal care time.

### Between-group variation in within-group regression by income level and living status

**Fig. 3:**
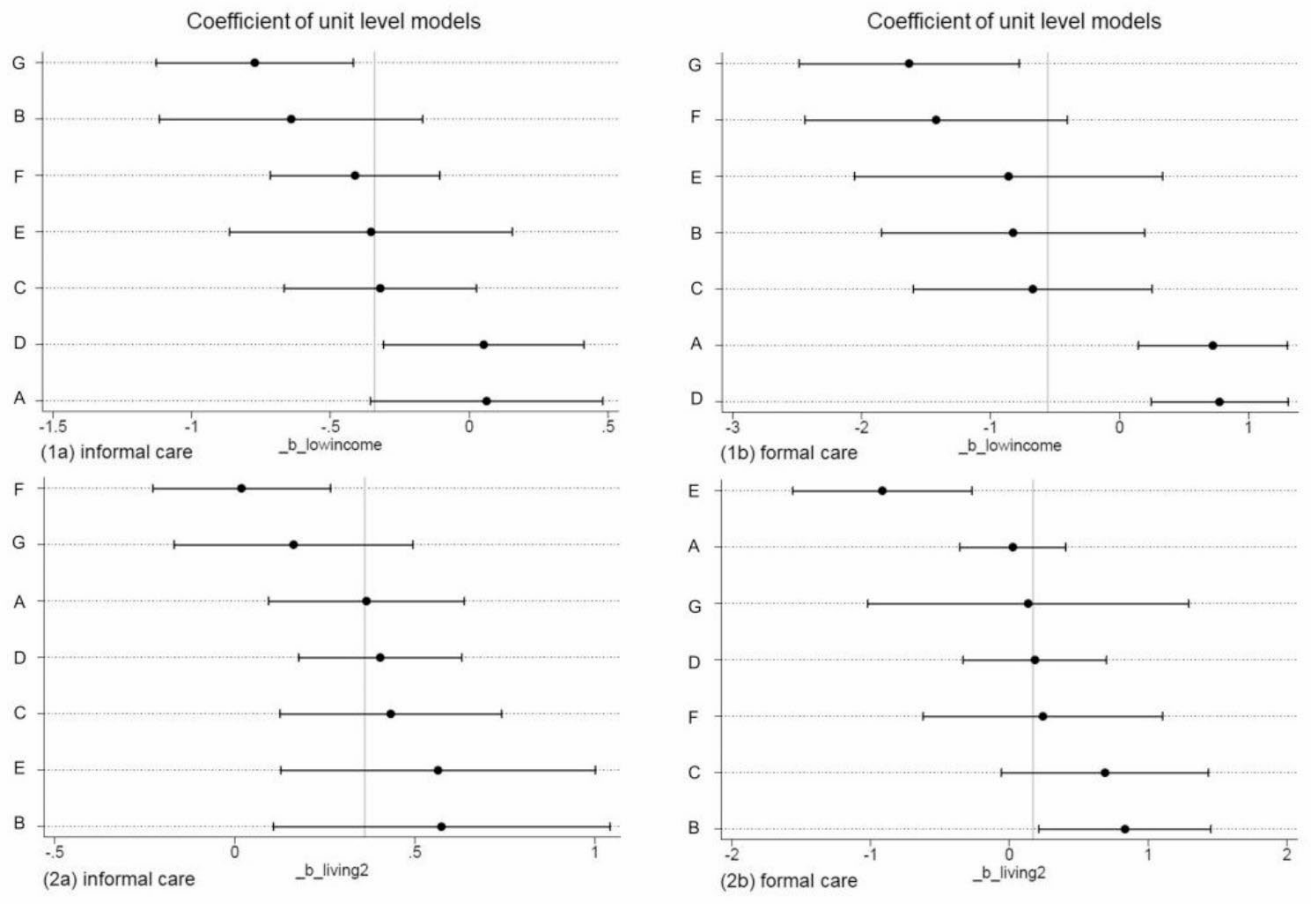
Between-group variation in within-group regression *Note*: (**1a**): Regression of low income on logged informal care time; (**1b**): Regression of low income on logged formal care time; (**2a**): Regression of living with family members on logged informal care time; (**2b**): Regression of living with family members on logged formal care time. Within-group regression estimates and 95% confidence intervals of care time are plotted in order of group-level care time

Figure 3 shows the between-group variation in within-group regressions. Significant differences in informal care use between older adults with low incomes and others existed in the fully dependent, limited-locomotion, and least dependent clusters. In the intact continence and vision cluster and fully dependent without impaired vision cluster, older adults who were poorer used more formal care. Apart from the limited-locomotion and least dependent clusters, older adults living with family members used more informal care than those in other living statuses. In the fully dependent cluster, older adults received more formal care when living with family members. In the cluster that was able to groom, older adults used less formal care when living with family members.

## Discussion

In this study, we identified seven functionally limited clusters among Chinese elderly claimants of public LTCI and examined the relationship between typologies of dependency and formal and informal care use by income and living situation. An important contribution of this study is the introduction of qualitative measurement of dependency, which expands the understanding of LTC use in older adults from the perspective of dependency cluster grouping. To our knowledge, this was the first study to examine and compare LTC use among typologies of dependency in the context of the Chinese LTCI.

Using cluster analysis, seven typologies of dependency were identified in the population: fully dependent without impaired vision, fully dependent, intact vision and transfer, intact continence and vision, able to groom, limited locomotion, and least dependent. Previous research using the same techniques has identified five functional limitation patterns among the community-dwelling US population using data from the Health and Retirement study: no difficulty, difficulty pushing/pulling, difficulty lifting, difficulty climbing stairs, and difficulty with the upper body [35]. Such discrepancies may be owing to population differences, as our study population included LTCI claimants, a potential physically disabled group of older people, which allowed us to put forth policy implications for LTCI.

Of the seven clusters, the fully dependent cluster had the most formal and informal care time per month; the least dependent cluster had the least formal and informal care time per month. These findings are consistent with previous studies, which showed that, on the whole, older people with severer dependency used more LTC [36]. Notably, by comparing the fully dependent cluster with the fully dependent without impaired vision cluster, we found that the former had more informal care time, but less formal care time than the latter. This supports a prior estimate that visual ability is an independent predictor of informal care hours received [37, 38]. Vision impairment increases dependence and also the risk of falls and injuries [39, 40], communication difficulties, and mental health problems [41, 42]. Elderly people with impaired vision need high-intensity nursing [37],varied equipment assistance [43], close attention to multimorbidity [44], and greater care adaption focusing on life satisfaction [45, 46]. For this population, living with family and receiving informal care may be the first choice because of their unspecified and unpredictable demands [47]. This also suggests that the formal care targeting visually impaired elderly people is insufficient due to the limited capacity of the network and associated copayment in some cases [38, 48].

In this study, we also descriptively compared the LTC use among typologies of dependency with different household characteristics. We observed a great disparity in care time for both formal and informal care between clusters in the low-income group; in the high-income group, these differences were very small. This implied that older adults with low household income may be more affected by their typologies of dependency. Household income was positively associated with greater health awareness [49], lower household financial burden [50], and larger social networks [51], suggesting advantages of receiving both formal and informal care. A case study in China found that older adults with sufficient or more financial means were more likely to exhibit a low/high need–high use pattern [52], which might lead to generally high utilization of LTC among older adults with different typologies of dependency. An empirical analysis of the medical services in Peninsular Malaysia has also shown that lower SES leads to lower demand for medical care, despite a greater “health need” (which may not be perceived as such) [49], implying that lower income levels may enhance the inequity of dependency. These findings suggest that the estimation of LTC demand and related utilization should take individual SES into account [53], and much consideration should be given to LTCI policies targeting the low-income elderly population.

When examining the inequality in terms of household income with respect to LTC use of clusters, we found a pro-poor inequality in formal care use in the fully dependent without impaired vision cluster and intact continence and vision cluster, contrasting with the findings of previous research [54]. This may be owing to earlier-implemented residual welfare policies in China, such as old age allowance, home-based assistance services, and various types of aid for poor families. These policies provide economic and service support to low-income elderly people, especially those living with disability [55]. We found a pro-wealthy inequality in formal care use in the least dependent cluster and limited-locomotion cluster; no income gradient was found in the fully dependent cluster, intact vision and transfer cluster, and the cluster able to groom. Possible explanations include that purchasing private domestic services in the market compensates for a lack of public eldercare among people with only moderate impairment [23]. Owing to their characteristics of need and a limited supply of formal care as mentioned above, family is the main source of care for elderly people who are fully dependent (with visual impairment) [47]; this leads to highly inelastic costs in terms of formal care time across income groups. Additionally, the popularization of adult diapers and promoted voiding (PV) provides a less time-intensive and affordable way to manage urinary incontinence, which may reduce the dependence on formal care and help with self-management, especially among elderly adults with good mobility and grooming ability [56].

With respect to informal care, inconsistent with prior studies [20], we found pro-wealthy inequality in the use of care among older adults in the least dependent cluster, limited-locomotion cluster, and the fully dependent cluster. This can be explained by the results of social network research. Higher SES is positively correlated with larger household size and a closer distance to children, pointing to an advantage in receiving informal care in households with higher income [57]. Concomitantly, the quality of social contacts was found to be higher in older middle-class people, with existing contacts translating into personal support more easily than for elderly working class adults [58]. However, we found that in other clusters, there was no difference between low-income older people and other older adults in the use of informal care. The cultural and moral context may play a counterweight role. Existing studies suggest that filial obligations may be stronger in groups with lower SES [59, 60], whose care arrangements are more strongly influenced by normative ideas than those of groups with higher SES [61, 62]. In particular, traditional Chinese culture has always valued filial piety and family care [63].

Previous research has shown that living status has an impact on inequalities in LTC use for older adults, as it might determine access to informal care or the ability to count on potential “advocates” in receiving LTC services [14, 24, 25]. In this study, we found that these effects worked in specific typologies of dependency, that is, living with family members was positively correlated with increased formal care time in the fully dependent cluster and with increasing informal care time generally except the least dependent cluster and limited-locomotion cluster. In particular, we speculated that informal care can serve as a substitute for formal care in LTC use among older adults of cluster able to groom. We found that living with family members decreased the likelihood of using formal care. Past studies have found this substitution effect exists as long as the disability level is low in elderly people, although this differs among countries [64, 65]. We did not directly assess the substitution of formal and informal care, but our findings help to improve understanding of this point from the perspective of dependency cluster grouping.

Existing studies do not provide a consensus regarding the inequality by SES in LTC use due to the mixed effect among individual health status and household conditions [17, 18, 36]. Our study contributes to this field of research by illustrating these inequalities across different typologies of dependency, which imply a distinction with respect to the potential mechanisms of LTC use in older adults. Older people with the least dependency and with limited locomotion are more vulnerable to the detrimental effect of low income on formal care use. Visual impairment and incontinence may have a more predictive power of the care arrangements. Pro-poor inequality and indifferent LTC use among other clusters seem to be explained by the context of Chinese institutions and culture. Older adults with full dependency benefit more from living with family members in terms of receiving formal care. Inequalities in an individual’s living status in terms of informal care use generally exist among older adults, except in those with the least dependency and limited locomotion.

These findings may inform the design and implementation of future interventions. For example, VI screening and early identification of VI among older adults are needed [66], which could facilitate early intervention and subsequent LTC. Targeting formal care should be considered for visually impaired older people. This study also provides some implications for the development of LTCI programs in China. Informal care is the safety net for homebound older adults in most typologies of dependency; therefore, policies should mainly pay attention to elderly people with low incomes who are living alone. Assistance for families and interventions such as respite service, care capacity building, and official leave for caregiving could be considered in the design of LTCI, especially for fully dependent (with impaired vision) older adults. Furthermore, family burden measurements may be applied as a screening tool to assess LTC needs, complementing the assessment of dependency [67].

Some limitations of this study should be mentioned. First, this was a cross-sectional study, which precluded inference regarding causality. Second, the study sample comprised potentially physically disabled older people and was not population-based. Therefore, these findings may not be generalizable to the whole population of older adults. Third, the role of external factors in LTC service utilization was not examined in this study. Caution should be exercised in comparing and explaining the LTC use of elderly individuals. Further research is needed to clarify the complex mechanism of LTC use using detailed longitudinal data covering individual and external factors. Fourth, while our empirical data supported the seven subgroups of typologies of dependency, and this result is similar with other research carried out in China using an alternative method (e.g. LCA) [68]; the seven typologies might be excessive for providing practical evidence. Still, we encourage future studies to explore the typologies of dependency using population data for different areas to contribute to a more concise and theoretically meaningful categorization.

## Conclusion

This study contributed to a better understanding of typologies of dependency and inequalities in LTC use among potentially physically disabled older people living at home in China. Our findings suggest that socioeconomic inequality in terms of household characteristics varies among typologies of dependency. These results could provide insights for researchers and policymakers to develop tailored LTC and targeted LTCI programs for older adults living at home and their family caregivers considering typologies of dependency and household conditions as a whole.

## Data Availability

The datasets analysed during the current study are not publicly available due to the restriction of data management but are available from the corresponding author on reasonable request.
